# Weekly Intraperitoneal Injection of Tamoxifen in an Inducible In Vivo Model of Junctional Epidermolysis Bullosa Generates Early and Advanced Disease Phenotypes

**DOI:** 10.1016/j.xjidi.2024.100331

**Published:** 2024-11-22

**Authors:** Eleri Mai Jones, Priya Garcha, Monique Aumailley, Edel Anne O’Toole, Emanuel Rognoni, Matthew Caley

**Affiliations:** 1 Cell Biology & Cutaneous Research, Blizard Institute, Queen Mary University of London, London, United Kingdom; 2 Centre for Biochemistry, Medical Faculty, University of Cologne, Cologne, Germany

**Keywords:** Inducible transgenic model, Junctional EB, Laminin, Skin inflammation

## Abstract

Junctional epidermolysis bullosa caused by loss-of-function variants in genes encoding the skin basement membrane proteins laminin 332, type XVII collagen, or integrin α6β4 affects patients from birth with severe blistering, eventually leading to scarring and early lethality. In this study, we have optimized a previously published junctional epidermolysis bullosa–knockout mouse model with weekly tamoxifen intraperitoneal injections, resulting in a more controllable and severe model. Owing to the titratable dosing, this model now recapitulates both early and advanced stages of the human disease, strengthening its use in therapeutic studies. The gradual loss of laminin-α3 in the skin of the mouse through weekly injections lead to generalized blistering and fibrotic dermal changes in multiple skin sites by week 12 after tamoxifen. Our findings demonstrate the usefulness of optimizing tamoxifen induction in Cre-loxP mouse models of extracellular matrix proteins, an approach that could be applicable to other emerging inducible transgenic disease models to improve their ability to mimic the human disease phenotype.

## Introduction

Junctional epidermolysis bullosa (JEB) is caused by pathogenic variants in genes encoding the skin basement membrane (BM) proteins laminin 332, type XVII collagen, or integrin α6β4 (Hammersen et al, 2016). Affected individuals suffer from blistering from birth leading to scarring and granulation tissue and susceptibility to infection. In generalized severe JEB, there is 50% mortality in the first 2 years of life often due to a failure to thrive or overwhelming infection (Petrof et al, 2022).

Several animal models for JEB and other epidermolysis bullosa subtypes exist (Bruckner-Tuderman et al, 2010); however, owing to severe blistering on the entire surface of the skin, most constitutive epidermolysis bullosa disease models are fatal perinatally, reducing their experimental utility for therapeutic development. Recent development of inducible conditional epidermolysis bullosa disease models where disease phenotypes can be induced postnatally are a significant step forward in the field. However, careful optimization of the disease phenotype in these in vivo models is required to allow them to fatefully mimic human disease progression and pathology. In 2017, the first adult inducible conditional knockout mouse model of JEB was published (Pesch et al, 2017). In this mouse model, the disruption of the *Lama3* gene is specifically induced in epidermal basal keratinocytes after crossing a *Lama3* flox/flox where *Lama3* exon 50–53 (murine equivalents of exons 49–52 of human *LAMA3* gene) is flanked by 2 loxP sites with the tamoxifen inducible Krt14CreERT2 line, expressing CreERT2 recombinase under control of the keratin 14 promoter (Pesch et al, 2017). By feeding a tamoxifen-supplemented diet from week 3 after birth, the JEB-epithelial knockout (JEB-eKO) mice (*Lama3*^flox/flox^K14^CreERT^) start to display moderate skin blistering after 10 weeks in mouse back skin and paws but were lacking many of the severe skin symptoms observed in patients with JEB leading to a complete loss of skin barrier function (Pesch et al, 2017). K14CreERT2 will also induce *Lama3* loss of function in keratin 14–positive epithelial cells of the oesophagus, stomach, and intestine. We speculated that a reason for the milder JEB phenotype leading to residual Lam-α3 observed in these JEB-eKO mice could be related to inefficient tamoxifen ingestion due to a progressive struggle to eat the tamoxifen-supplemented diet. In this study, we have optimized and characterized the previously published JEB-eKO model with weekly tamoxifen intraperitoneal injections, which results in a more controlled and severe phenotype of JEB, with blistering observed across all body sites. By carefully titrating the tamoxifen dosage, this optimized JEB-eKO model is now able to faithfully recapitulate the early and advanced key features of human JEB disease, strengthening its translational potential to discover previously unreported disease mechanisms and targets. We believe that a similar approach could be applicable to other emerging inducible transgenic disease models in skin and other organs to improve their ability to mimic the human disease phenotype.

## Results and Discussion

### Optimizing JEB phenotype induction in mouse skin

**Figure 1:**
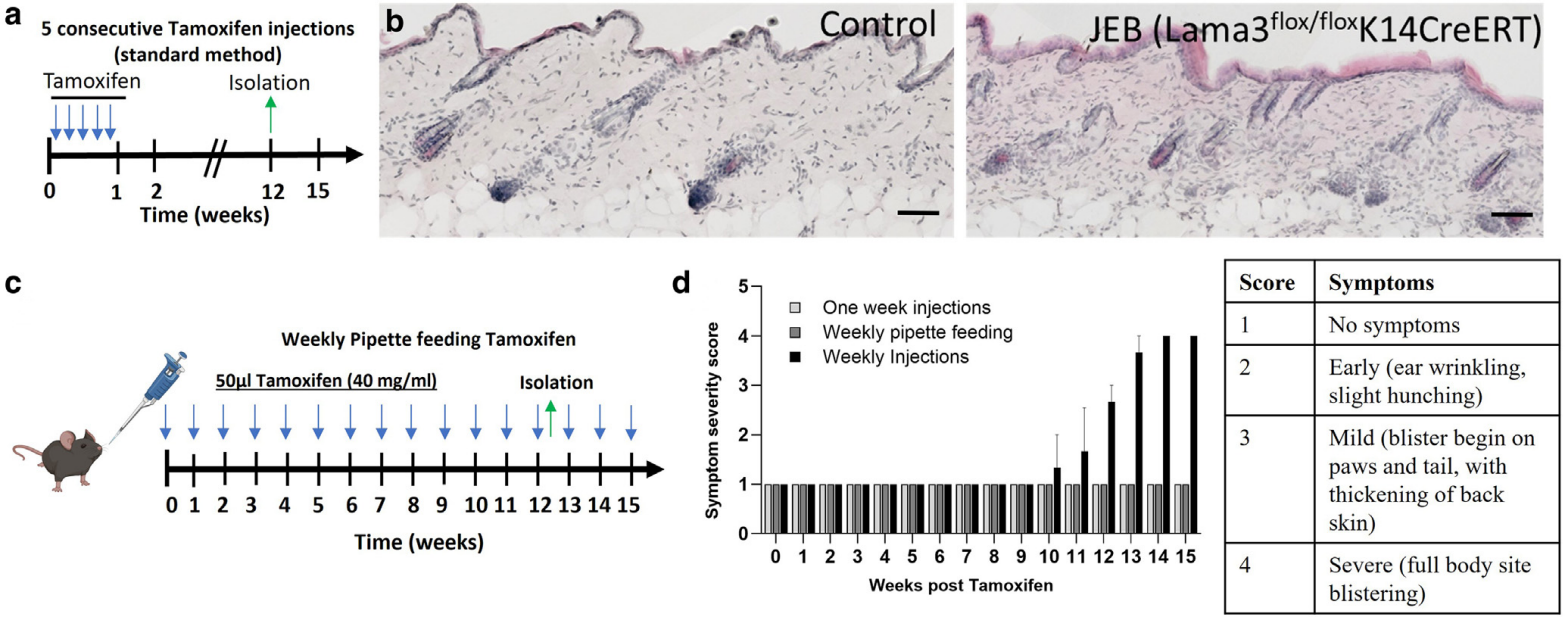
**Tamoxifen optimization of JEB-eKO (Lama3**^**flox/flox**^**/K14**^**CreERT**^**) mouse.** (**a**) Experimental design shows standard method of tamoxifen induction. (**b**) Skin histology sections 12 weeks after initial 5 consecutive doses of tamoxifen showing no difference between control and JEB-eKO skin Scale bar = 40μm. (**c**) Experimental design for weekly pipette feeding of tamoxifen. (**d**) Graph shows symptom severity comparison of all 3 tamoxifen-dosing treatments tested, whereas table indicates what symptom correlates to each score between 1 and 4. JEB-eKO, junctional epidermolysis bullosa–knockout.

In the process of optimizing the tamoxifen dosing in the JEB-eKO mice, we first tested weekly pipette feeding of tamoxifen dissolved in corn oil for up to 15 weeks, leading only to a mild JEB skin phenotype beginning to show at the end of those 15 weeks (Figure 1c and d). We then tested a common intraperitoneal tamoxifen administration protocol (5 daily consecutive injections of 100 μl tamoxifen [20 mg/ml]) (The Jackson Laboratory, 2011), which failed to induce a visible JEB phenotype by 12 weeks owing to inefficient *Lama3* deletion (Figure 1a–c).

**Figure 2:**
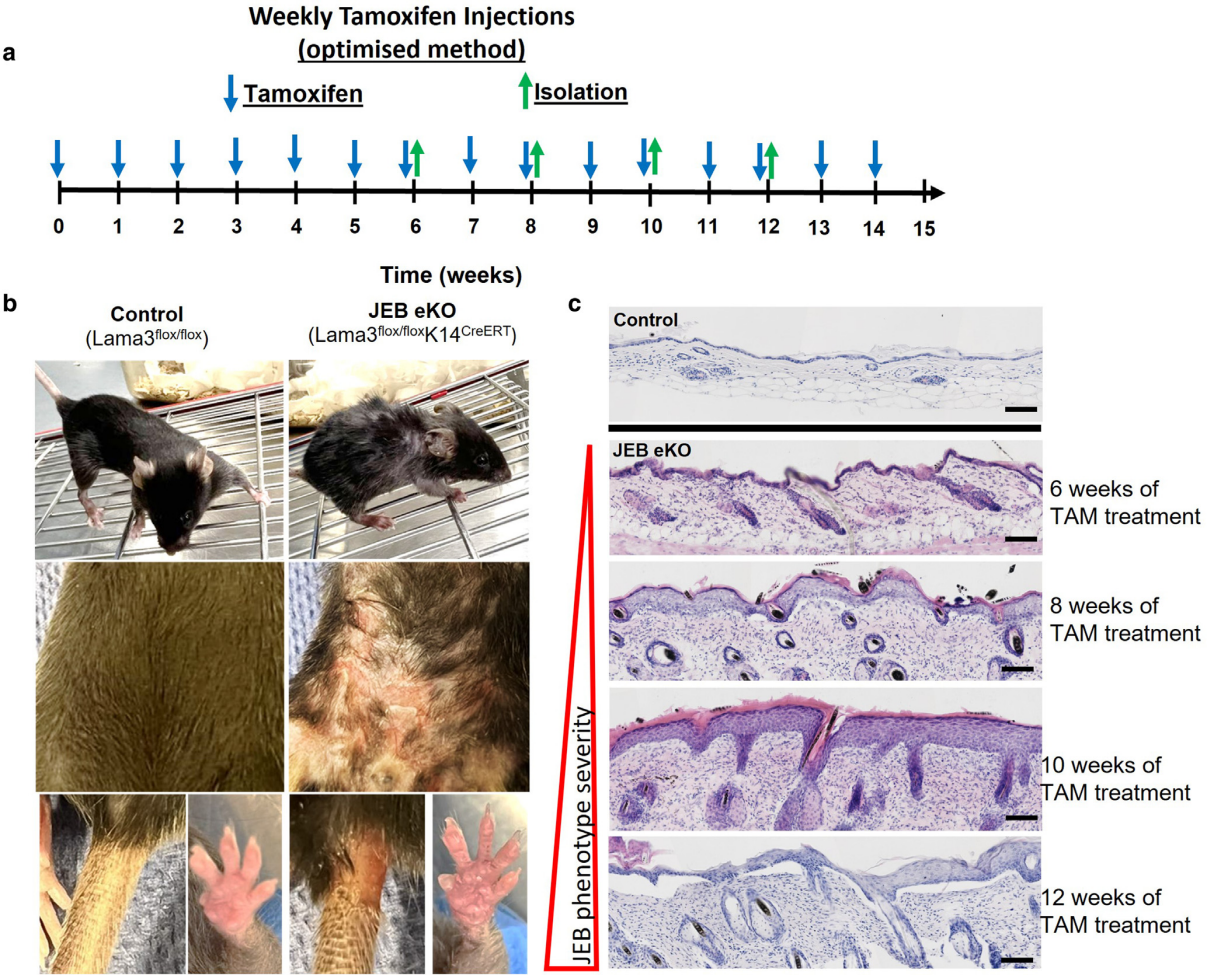
**JEB-eKO (Lama3**^**flox/flox**^**/K14**^**CreERT**^**) mouse during weekly TAM treatment.** (**a**) Experimental design shows TAM-induction strategy and skin isolation time points to follow JEB severity after induction. (**b**) Overview images showing control mouse and the JEB mouse model after 12 weeks of TAM treatment (n = 3). The JEB-eKO mouse has a severe JEB phenotype, appears smaller, and displays a hunched posture. Blisters are observed on the skin, tail, and paws. (**c**) H&E images of mouse back skin allowing the comparison between control and the JEB-eKO mild (weeks 6–8 TAM) and severe (weeks 10–12 TAM) mouse model over the course of TAM treatment. Bar = 50 μm. JEB, junctional epidermolysis bullosa; JEB-eKO, junctional epidermolysis bullosa–knockout; TAM, tamoxifen.

**Figure 3:**
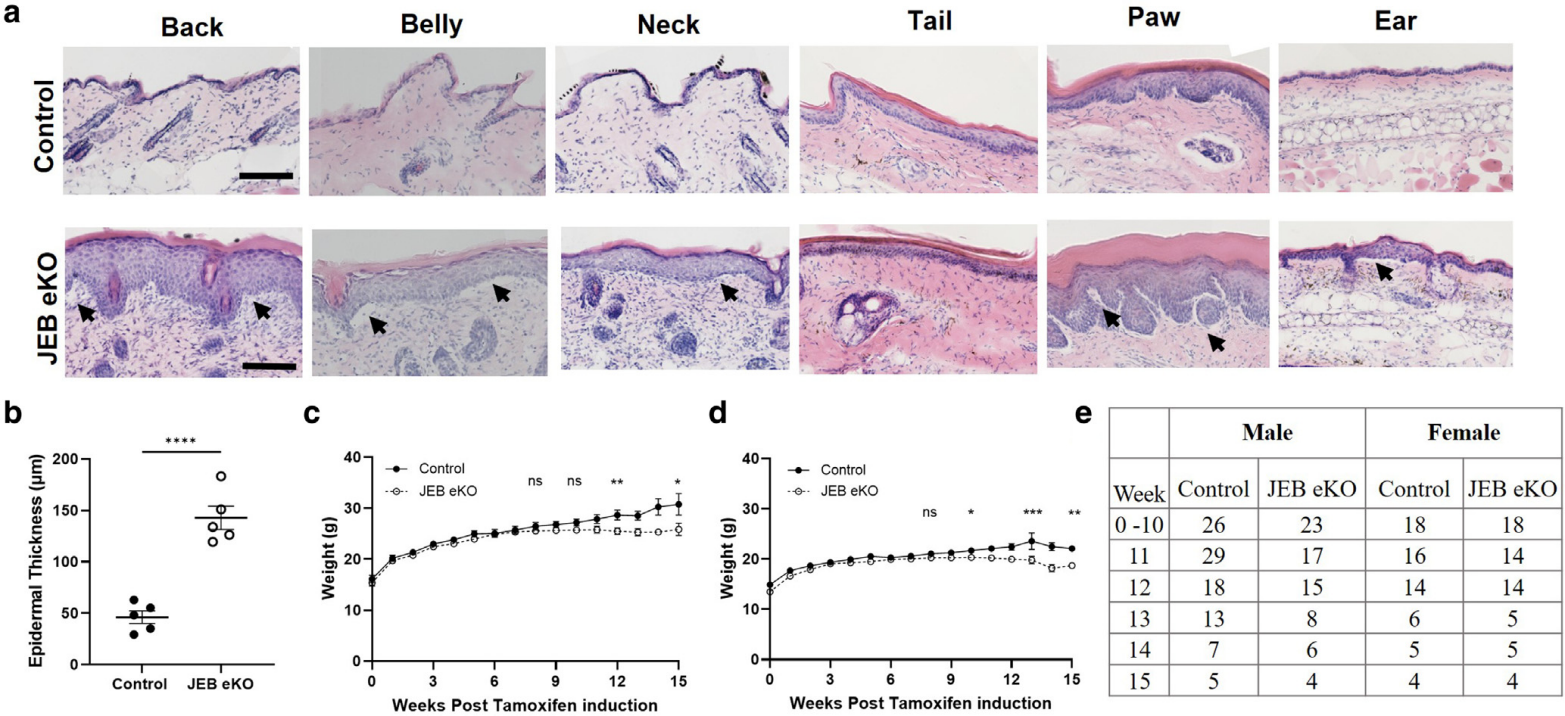
**Further characterization of JEB-eKO (Lama3**^**flox/flox**^**/K14**^**CreERT**^**) mouse.** (**a**) H&E staining of skin samples from various body sites 12 weeks after tamoxifen. All JEB-eKO samples show a thickened epidermis with high immune infiltration along with visible blisters in back, belly, paw pad, and ear (indicated by arrows). (**b**) Quantification of back skin epidermal thickening 12 weeks after tamoxifen in JEB-eKO and control mice (n = 5 biological replicates, and mean ± SEM of 10 measurements per mouse is shown). Statistical analysis was performed using *t*-test with Mann–Whitney correction. ∗∗∗∗*P* < .0001. (**c, d**) Weight development during tamoxifen treatment. (**c)** Males; (**d)** females. Twelve and 15 week after tamoxifen, both male and female mice start to weigh significantly less than the control. (**e)** Table shows the numbers of biological replicates used for weight graphs (in **c** and **d**) over 0–15 weeks of tamoxifen treatment. Statistical analysis was performed using 2-way ANOVA shows significance at ∗*P* < .05, ∗∗*P* < .01, ∗∗∗*P* < .001, and ∗∗∗∗*P* < .0001. Bar = 100 μm. JEB-eKO, junctional epidermolysis bullosa–knockout.

Optimized induction of weekly tamoxifen injections (100 μl of 20 mg/ml) greatly improved the phenotype and severity of JEB owing to efficient loss of Lam-α3 in the skin of the mouse. Weekly injections resulted in consistent gradual loss of BM Lam-α3, leading to generalized blistering and fibrotic dermal changes in the back, belly, paw, and tail skin by week 12 after tamoxifen (Figures 1c, 2, and 3). In the previous study by Pesch et al (2017), blisters were only noted on the back skin and the foot pad/paw. Initial epidermal thickening and fibrotic dermal changes became visible after 6 weeks of tamoxifen treatment. By week 10, there is significant epidermal thickening (Figure 2b) along with high immune cell infiltration and skin blistering in multiple body regions. Mice developed a hunched posture, showed reduced front paw grip strength, and started to lose weight. At week 12 of treatment, JEB-eKO skin displayed large blistering wounds (Figures 2 and 3c and d). The requirement for continuous tamoxifen treatment to maintain deletion of BM Lam-α3 suggests a recovery of laminin-expressing keratinocytes from other sources that were previously keratin 14 negative triggered by the micro wounds in the JEB-eKO skin. Cellular plasticity in the wound environment has previously been described with at least 3 different sources of new skin cells observed in response to wounding: dedifferentiation of keratin-negative Gata6+ cells (Donati et al, 2017), sebaceous duct cells (Kurita et al, 2018), fibroblast mesenchymal–epithelial transition, and myeloid cell differentiation (Guerrero-Juarez et al, 2019).

### Cellular and extracellular matrix changes in JEB-eKO mice

**Figure 4:**
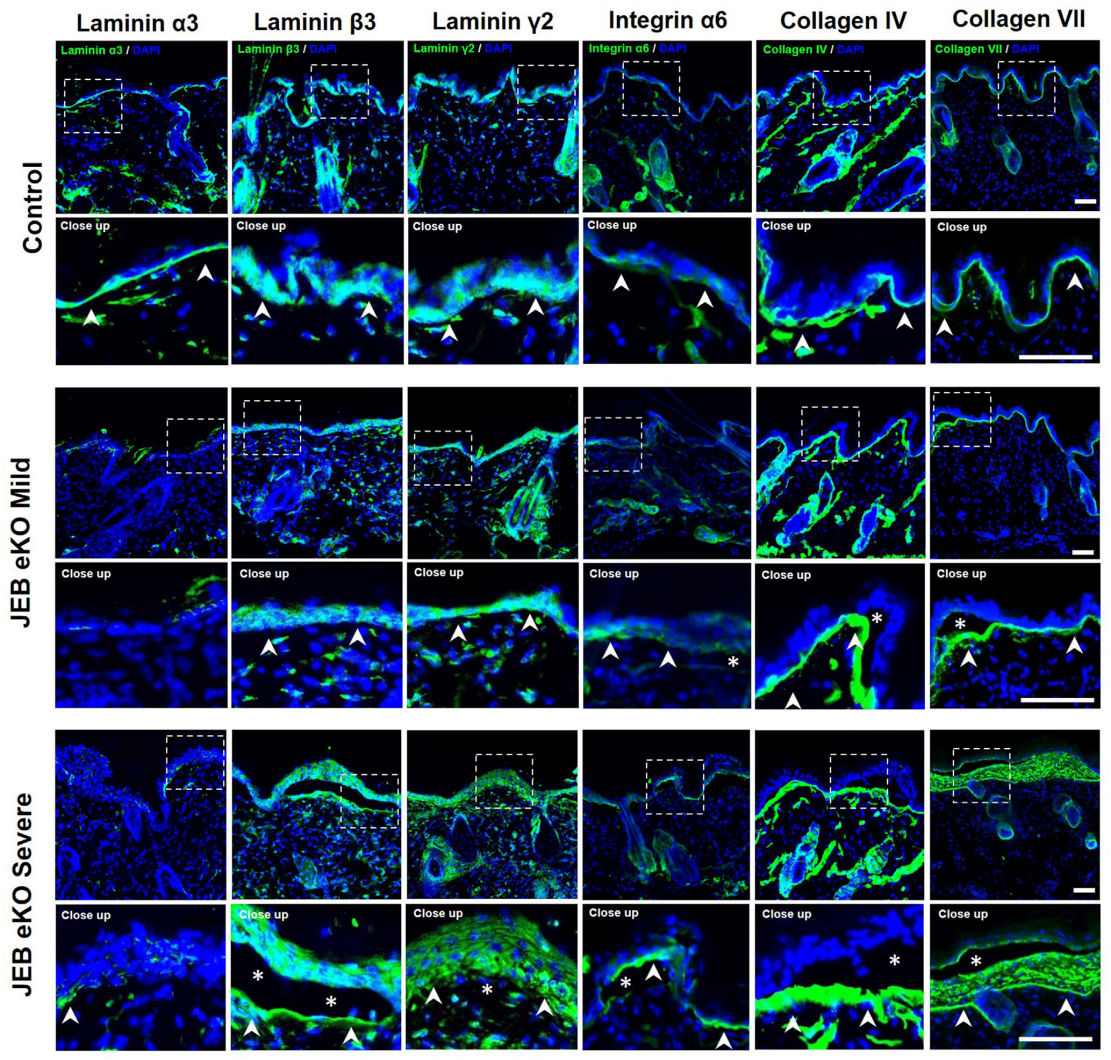
**Basement membrane characterization of mild and severe JEB-eKO (Lama3**^**flox/flox**^**/K14**^**CreERT**^**) mouse skin.** Shown is immunofluorescence staining of basement membrane proteins laminin-α3, laminin-β3, laminin-γ2, integrin-α6, collagen IV, and collagen VII in control mice and JEB-eKO mice with mild and severe back skin sections. Close up of boxed area is shown below each image. The asterisk (∗) indicates blister area; arrow heads point to location of the basement membrane. Bar = 50 μm. JEB-eKO, junctional epidermolysis bullosa–knockout.

**Figure 5:**
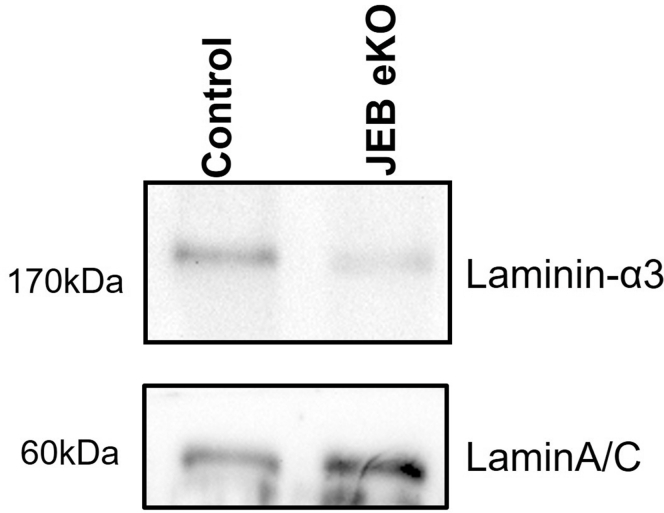
**Loss of laminin-α3 in JEB-eKO mouse epidermis.** Western blot analysis of laminin-α3 in both control and JEB-eKO mouse skin was performed. Lamin A/C was used as an internal control of protein loading. JEB-eKO, junctional epidermolysis bullosa–knockout.

**Figure 6:**
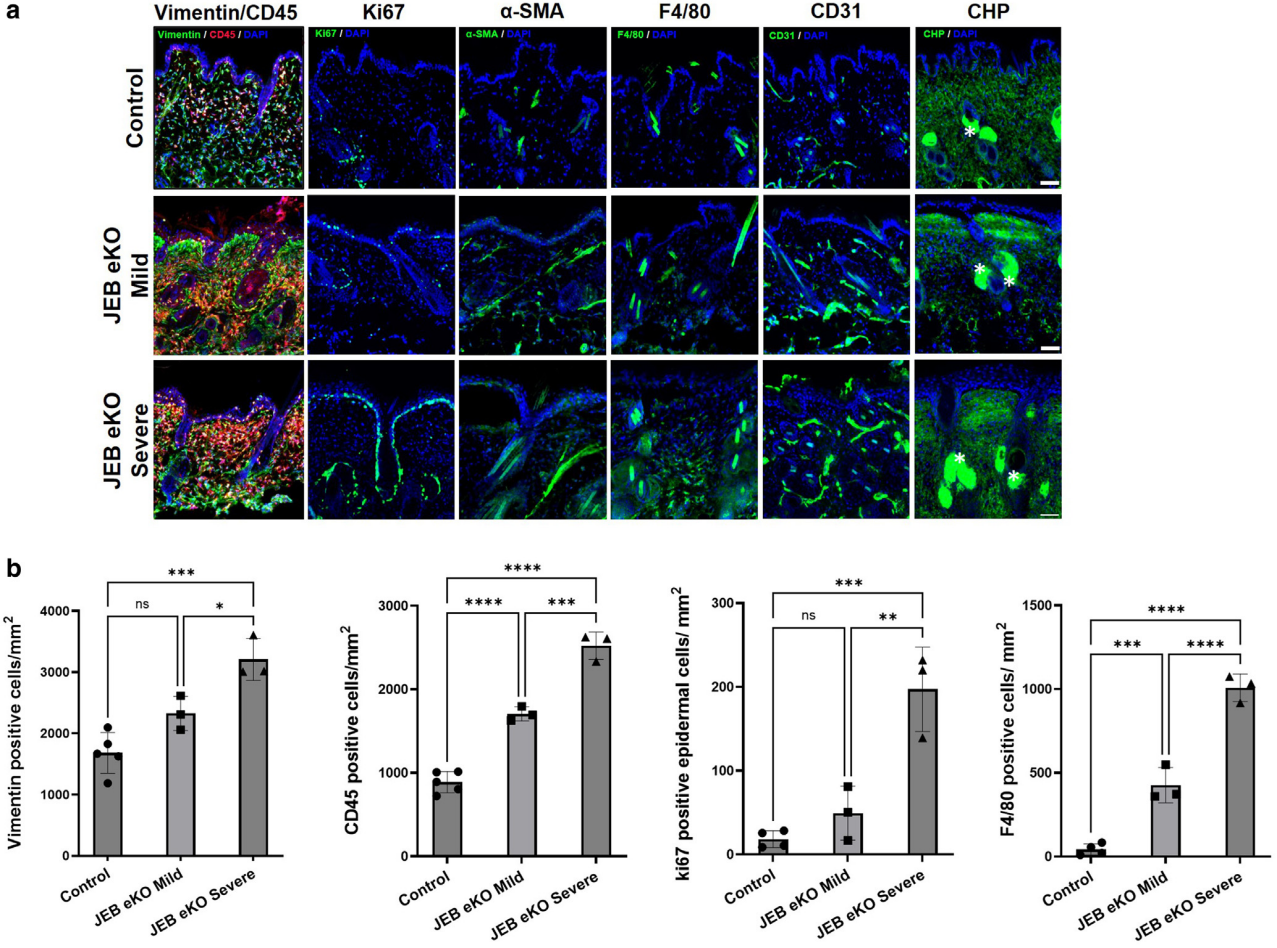
**Cellular and ECM changes in JEB-eKO (Lama3**^**flox/flox**^**/K14**^**CreERT**^**) mouse skin.** (**a**) Further characterization of fibroblasts (vimentin, green), immune infiltration (CD45, red, and macrophages by F4/80), proliferation (Ki-67), αSMA, blood vessels (CD31), and new collagen matrix deposition (CHP). The asterisk (∗) indicates unspecific staining. Bar = 50 μm. (**b**) Quantification in control (n = 4–5 biological replicates showing mean ± SEM) and in JEB-eKO mice with mild (n = 3 biological replicates showing mean ± SEM) and severe (n = 3 biological replicates showing mean ± SEM) disease of fibroblast (Vim+), immune cell (CD45+ and macrophages by F4/80), and epidermal proliferation (Ki-67+ basal cells). Statistical analysis was performed using 1-way ANOVA with multiple comparisons showing significance at ∗*P* < .05, ∗∗*P* < .01, ∗∗∗*P* < .001, and ∗∗∗∗ *P* < .0001. Bar = 100 μm. αSMA, α-smooth muscle actin; CHP, collagen hybridizing peptide; ECM, extracellular matrix; JEB-eKO, junctional epidermolysis bullosa–knockout.

Immunofluorescence staining and western blot analysis confirmed loss of BM Lam-α3 (Figures 4 and 5) in JEB-eKO mice with early and advanced JEB phenotype. There was a decrease in Lam-β3 and Lam-γ2 staining at the BM, with the staining becoming intracellular and much more diffuse with increased severity in JEB-eKO mice (Figure 4). Expression of other BM proteins increased with severity, including integrin-α6, collagen IV, and collagen VII. Collagen VII specifically increased within blistered areas in JEB-eKO mice with severe disease, indicative of continuous tissue remodeling (Figure 4). Comparing early (mild) with advanced (severe) JEB phenotypes revealed a progressive increase in fibroblasts (CD45−Vim+) and immune cells (CD45+Vim−), such as macrophages (F4/80+), especially in the upper dermis closer to site of blistering (Figure 6). Notably, although keratinocytes are highly proliferative around small blisters in early (mild) JEB-eKO skin, this is lost in large blisters, and there is an increase in myofibroblasts (α-smooth muscle actin–positive) and fibrotic extracellular matrix remodeling (increased collagen hybridizing peptide [CHP] staining) as well as an increase in blood vessel (CD31+) formation in the upper dermis at the advanced JEB disease stage (Figure 6), suggesting a progressive impairment of the skin’s regenerative capacity and fibrotic progression, which warrant further investigation.

Inducible disease mouse models do not always reflect all key features of the human disease. In this study, we show an improved, titratable, and highly controlled method of inducing disease in a tamoxifen-inducible Cre-loxP system. Specifically, in this JEB-eKO model, we see development of blisters over time and can follow disease progression from mild to severe, with exacerbation of whole-body disease phenotype (Figure 3). We believe that the previously published JEB/Lama3-knockout mouse phenotype is comparable with what we refer to as our mild phenotype after 8–10 weeks of tamoxifen. Previously, mice were culled after 15 weeks of tamoxifen (Pesch et al, 2017); however, we can very rarely keep our mice alive that long because the phenotype is too severe after 12 weeks of tamoxifen. Thus, our findings demonstrate the usefulness of carefully optimizing the method for tamoxifen induction in Cre-loxP mouse models, which could be relevant for other inducible Cre-loxP disease mouse models in skin and other organs.

## Materials and Methods

### Animals and treatment

Mice were crossed to produce offspring of both *Lama3*^flox/flox^K14^CreERT^ (JEB-eKO) and *Lama3*^flox/flox^ (control) genotype. At the age of 3 weeks, mice were weaned and began tamoxifen treatment. Mice were treated interperitoneally with 100 μl (80 mg/kg of body weight) tamoxifen dissolved in corn oil once a week for up to 15 weeks. During optimization, mice were also treated daily for 5 consecutive days with 100 μl intraperitoneal tamoxifen injections (80 mg/kg of body weight) or by pipette feeding weekly with 50 μl (40 mg/ml tamoxifen in corn oil). Tamoxifen was dissolved in corn oil (20 mg/ml) by intermittent water bath sonication for 10 minutes. Mice were weighed weekly during tamoxifen treatments to check for any adverse weight loss. Mice were killed by carbon dioxide asphyxiation or cervical dislocation. All efforts were made to minimize suffering for the mice. Skin tissue from multiple body sites were harvested for further analysis. Tissue was collected at indicated time points after tamoxifen treatment and embedded into optimal cutting temperature compound.

### Histochemical and immunostaining

Mouse tissue samples were embedded in optimal cutting temperature compound (Life Technologies) prior to sectioning. Cryosections of 12-μm thickness were fixed with 4% paraformaldehyde (10 minutes at room temperature). For H&E staining, after fixation, sections were processed through a series of gradient alcohols to water (100% ethanol [EtOH], 100% EtOH, 95% EtOH, 70% EtOH, distilled eater; 5 minutes each). Slides were stained for 5 minutes in Mayer hematoxylin solution and then washed in running tap water for 10 minutes, followed by 5 rinses in 1% acid alcohol. Sections were counterstained in eosin solution for 2 minutes and then dehydrated through 70, 90, and 100% EtOH (5 minutes each). To finish, sections were mounted in DPX mounting medium, and images were obtained.

For immunostaining, after fixation, sections were permeabilized with 0.1% Triton X-100/PBS (10 minutes at room temperature), blocked with 5% BSA/PBS (1 hour at room temperature), and stained with the primary antibodies to laminin-α3 (1:200, kindly provided by Prof Aumailley), laminin-β3 (1:200, clone PA5-21514, Invitrogen), laminin-γ2 (1:200, clone PA5-79578, Invitrogen), integrin-α6 (1:200, clone GoH3, BioLegend), collagen IV (1:200, Acris), collagen VII (1:500, clone NC1, gift by Prof Mei Chen), vimentin (1:200, clone D21H3, Cell Signaling Technology), CD45 (1:200, clone 30-F11, eBioscience), Ki-67 (1:200, Abcam, ab15580), α-smooth muscle actin (1:200, Abcam, ab5694), F4/80 (1:200, clone BM8, BioLegend), and CD31 (1:200, clone 390, BioLegend) overnight at 4 °C. Sections were washed in PBS and labeled with secondary antibodies (all 1:250, Alexa Fluor 488, A-32731, Invitrogen; Alexa Fluor 555, A-21434, Life Technologies) and DAPI (1 mg/ml stock solution diluted 1:50,000 in PBS, D1306, Thermo Fisher Scientific) or 1 hour at room temperature with at least 3 PBS washes in between. Sections were mounted with immumount and covered with a coverslip before imaging.

For the CHP, 12-μm cryosections of upper back skin were thawed, fixed with 4% paraformaldehyde (10 minutes at room temperature), permeabilized with 0.1% Triton X-S (10 minutes at room temperature), blocked with 5% BSA/PBS (1 hour at room temperature), and stained with CHP biotin conjugate (BIO300, 3Helix). The biotin conjugate CHP was diluted in 5% BSA/PBS (1:200), and according to manufacturer’s instructions, the biotin conjugate CHP probe was heated for 5 minutes at 80 °C and then quenched on ice for 90 seconds before applying to the tissue sections and incubating overnight at 4 °C. Sections were washed 3 times and incubated with streptavidin-Alexa Fluor 555 (1:1000) (S32355, Thermo Fisher Scientific) and DAPI (1:1000) for 1 hour at room temperature. After an additional 3 washes, slides were mounted as described earlier.

### Skin protein isolation and western blotting

Protein was isolated from mouse epidermis by placing whole-mouse skin, previously shaved to remove hair, in 1x trypsin for 1 hour at 37 °C (epidermis side down). The epidermis was then easily separated from the dermis before being diced into smaller sections and homogenized. Epidermal pieces were submerged in DMEM media before centrifugation at 13,000*g* at 4 °C. Supernatant was discarded, and cell pellet was resuspended in 1× RIPA buffer supplemented with a protease inhibitor cocktail (Roche).

For immunoblot analysis, mouse epidermal lysates were subjected to 7% SDS-PAGE. The presence of protein was detected by immunoblotting using a laminin-α3 (Abcam, ab11575) and laminA/C (Santa Cruz Biotechnology, sc376248) primary antibody at 4 °C overnight and a horseradish peroxidase–coupled antimouse or antirabbit secondary antibody for 1 hour at room temperature and developed using Enhanced Chemi Luminescence.

### Image analysis and quantification

All images (including H&E) were generated using the TissueFAXS Plus microscope (Tissue Gnostics). All images were processed using TissueFAXS software and FIJI ImageJ software. Images were quantified using QuPath software. One-way ANOVA with multiple comparison statistical analysis was performed on quantified images and plotted using GraphPad.

## Ethics Statement

All animal experiments were subject to local ethical approval and performed under the terms of a United Kingdom government Home Office licence (P48019841).

## Data Availability Statement

Primary research data can be obtained upon request by contacting MC (m.caley@qmul.ac.uk).

## ORCIDs

Eleri Mai Jones: http://orcid.org/0000-0001-7022-987X

Priya Garcha: http://orcid.org/0009-0006-6449-0872

Monique Aumailley: http://orcid.org/0000-0002-4578-0966

Edel Anne O’Toole: http://orcid.org/0000-0002-4084-4836

Emanuel Rognoni: http://orcid.org/0000-0001-6050-2860

Matthew Caley https://orcid.org/0000-0003-0504-9922

## Conflict of Interest

The authors state no conflict of interest
